# Inflammation and Starvation Affect Housekeeping Gene Stability in Adipose Mesenchymal Stromal Cells

**DOI:** 10.3390/cimb46010054

**Published:** 2024-01-19

**Authors:** Enrico Ragni, Simona Piccolo, Michela Taiana, Caterina Visconte, Giulio Grieco, Laura de Girolamo

**Affiliations:** Laboratorio di Biotecnologie Applicate all’Ortopedia, IRCCS Istituto Ortopedico Galeazzi, Via Cristina Belgioioso 173, 20157 Milano, Italy; enrico.ragni@grupposandonato.it (E.R.); simona.piccolo@grupposandonato.it (S.P.); michelamaria.taiana@grupposandonato.it (M.T.); giulio.grieco@grupposandonato.it (G.G.); laura.degirolamo@grupposandonato.it (L.d.G.)

**Keywords:** mesenchymal stromal cells, housekeeping genes, inflammation, starvation, regenerative medicine, osteoarthritis, secretome

## Abstract

Due to the scientific success of in vitro and in vivo model studies, the interest in using mesenchymal stromal cells (MSCs) for the treatment of orthopaedic conditions is growing. In the context of osteoarthritis (OA), MSCs, and, in particular, those derived from adipose tissues (ASCs), have found broader access to clinical use as active components of minimally manipulated orthobiologics, as well as clinically expanded cell preparations, or to collect their released factors (secretome) for cell-free approaches. In this regard, while both inflammatory priming and starvation are common strategies used to empower cell potency or collect the secretome, respectively, little is known about the possible influence of these approaches on the stability of housekeeping genes (HKGs) for molecular studies able to fingerprint cell phenotype or potency. In this report, the reliability of five commonly used HKGs (*ACTB*, *B2M*, *GAPDH*, *HPRT1* and *RPLP0*) was tested in ASCs cultured under standard protocol after inflammatory priming or starvation. Gene expression data were computed with four different applets able to rank genes depending on their stability in either single or combined conditions. The obtained final ranking suggests that for each treatment, a specific HKG is needed, and that starvation is the condition with the stronger effect on HKGs’ stability and, therefore, reliability. The normalization effect of proper HKGs’ use was then validated on three genes involved in OA and whose product is released by ASCs. Overall, data presented herein confirm that the choice of the best HKG has to be carefully considered and that each specific condition has to be tested to identify the most reliable candidate.

## 1. Introduction

Osteoarthritis (OA) is a disease of a whole joint as an organ, and it may affect the synovium, the capsule, the articular cartilage, the subchondral bone and the ligaments [1]. The most evident symptoms associated with OA are degenerative changes, coupling synovial inflammation (synovitis) and cartilage sufferance in a feedback-loop mechanism. The main goal in OA therapeutic research is restoring the articular homeostasis, especially in early stages of the pathology, in order to delay or possibly halt cartilage degeneration and postpone or even avoid possible joint replacement.

Anti-inflammatory and pro-regenerative abilities are, therefore, crucial characteristics of new products envisioned as alternatives to traditional approaches to OA treatment. In this regard, orthobiologics emerged as a valuable option in clinical practice [2] to modulate the pathological microenvironment toward a more physiological one by interacting with tissue-resident cells. Among different orthobiologics, those based on bone marrow and adipose tissue have gained popularity due to their efficacy in educating the microenvironment towards a pro-regenerative effect mediated by mesenchymal stromal cells (MSCs) [3]. In particular, adipose-MSCs (ASCs) were demonstrated to exert anti-inflammatory and tissue homeostatic actions, either as the bioactive pillar of minimally manipulated products [4], such as stromal vascular fraction (SVF) or micro-fragmented adipose tissue (mFAT), or as clinical-grade expanded products [5], including cells and cell-free soluble factors/extracellular vesicles (EVs) termed “secretome”. Of note, a similar bio-active function was demonstrated for bone marrow-MSCs (BMSCs), again as the leading actor of minimally manipulated products, such as bone marrow aspirate concentrate [6], or as GMP preparations [7]. To date, no conclusive data about ASCs or BMSCs superiority are available, although the ease of tissue harvest and higher number of embedded MSCs [8] suggest adipose-derived products as a preferable option.

ASCs pro-regenerative and anti-inflammatory abilities were shown to be empowered through direct interaction with the diseased environment [9], as the inflamed joint, and recapitulated in vitro through cell priming [10], which is a common approach to the generation of cell-based or cell-free products with improved potential. For the latter, whole secretome or EVs, serum starvation is a common procedure [11,12], resulting in a futuristic alternative to cell-based therapy. Nevertheless, both priming and starvation have a great impact on the treated cells, and a deep understanding of the influence of these approaches is crucial from the perspective of a reliable molecular characterization for the clinical translation of ASC-based products. Following this archetype, some studies already investigated the stability of commonly used housekeeping genes (HKGs) in ASCs, with special focus on proliferation and differentiation [13,14,15,16], showing how culturing conditions may dramatically affect reference gene reliability.

In this work, we aimed to expand knowledge about ASCs-HKGs by investigating the stability of five commonly used normalizers in cells cultivated under different conditions mimicking those used for orthopaedic research and therapeutic approaches such as the following: standard conditions in bovine serum without and with inflammation mimicking the inflamed joint environment, starvation after meeting the two above-mentioned conditions to produce cell-free secretomes, or starvation in the presence of inflammatory mediators to isolate empowered clinical-grade EVs. Data were computed by four applets recognized by the scientific community to rank the stability of putative HKGs, and the results were validated on gene coding for factors contributing to ASCs potential in OA-related clinical applications.

## 2. Materials and Methods

### 2.1. Ethics Statement

The study was performed under the Institutional Review Board approval and Informed Consent administration to patients (San Raffaele Hospital Ethics Committee, approved on 16 December 2020, registered under number 214/int/2020), following the 1964 Helsinki declaration and its later amendments or comparable ethical standards.

### 2.2. Adipose Mesenchymal Stromal Cells (ASCs) Isolation and Expansion

Waste adipose tissue of three female donors (median 32 yo ± 3) undergoing elective plastic surgery was digested for 30 min at 37 °C with 0.075% *w*/*v* type I collagenase (Worthington Biochemical Co., Lakewood, NJ, USA). A 100 µm cell strainer was used to filter digested tissue, and the flow-through was centrifuged (1000× *g*, 5 min) to recover cells that were seeded at 5 × 10^3^ cells/cm^2^ in DMEM + 10% FBS (GE Healthcare, Piscataway, NJ, USA) and 1% L-glutamine plus penicillin–streptomycin (Life Technologies, Carlsbad, CA, USA). Cells were cultivated at 37 °C, 5% CO_2_ and 95% humidity. Experiments were performed with cells at two passages, whose morphology was evaluated using imaging analysis with an Olympus CKX41 microscope (Olympus, Tokyo, Japan). When ASCs reached 90% confluence, five conditions were set up: (i) 48 h in complete medium (DMEM + 10% FBS), hereafter named condition “F”; (ii) as condition F but supplemented with 1 ng/mL interleukin 1 beta (IL1B), “F+IL1”; (iii) after 48 h in condition F, cells were washed three times with saline solution and maintained in DMEM without serum for 48 h for starvation condition, “S”; (iv) as condition S but supplemented with 1 ng/mL IL1B, “S+IL1”; (v) after 48 h in condition F+IL1, cells were washed three times with saline solution, and starvation (DMEM) without serum was maintained for 48 h, “F+IL1/S”. At the end of treatments, cells were recovered for viability test, performed with a NucleoCounter^®^ NC-3000™ (ChemoMetec, Allerod, Denmark), flow cytometry analysis or RNA extraction.

### 2.3. Flow Cytometry Analysis

Flow cytometry was used to score positive or negative MSC or hemato/endothelial markers (antibodies: CD34-PE Vio770 clone AC136, CD73-PE clone REA804, CD90-FITC clone REA897, CD105-PerCP Vio700 clone REA794 and CD45-PE Vio770 clone REA747; Miltenyi Biotec, Bergisch Gladbach, Germany. CD44-PerCP clone 44PP2; Immunostep, Salamanca, Spain) with a CytoFLEX flow cytometer (Beckman Coulter, Fullerton, CA, USA), collecting a minimum of 30,000 events. CytExpert v2.3 (Beckman Coulter) software was used to process data. Antibodies were used in the following combinations: CD73/90/105/45 and CD34/44.

### 2.4. RNA Extraction and mRNA Profiling

RNA was purified with the miRNeasy Micro Kit (Qiagen, Hilden, Germany) following the manufacturer’s protocol. After quantification, equal amounts of purified RNA for each sample were retrotranscribed with RT^2^ First Strand Kit (Qiagen) according to the protocol provided by the manufacturer. The reaction included an internal reverse transcription control (RTC) to monitor efficiency in cDNA synthesis. cDNA was supplemented with RT^2^ SYBR Green qPCR Mastermix (Qiagen) and stored at −20 °C. For all samples, to ensure homogeneity of amplification, a final concentration of 0.8 ng (initial RNA)/µL was used. SYBR green included a positive PCR control (PPC) to monitor efficiency of PCR amplification reactions performed with QuantStudio™ Real-Time PCR system (Thermofisher Scientific, Waltham, MA, USA). Each sample was analysed in technical duplicate with the QuantStudio™ 12K Flex Design & Analysis Software v2.7.0. Amplification was considered present with Ct < 35. Validated assays tested for real-time PCR performance for specificity and amplification efficiency (>90%), resulting in a single product of the correct size, were purchased from Qiagen: Actin B (*ACTB*, code: PPH00073G), Beta 2 microglobulin (*B2M*, PPH01094E), Glyceraldehyde-3-phosphate dehydrogenase (*GAPDH*, PPH00150F), Hypoxanthine phosphoribosyltransferase 1 (*HPRT1*, PPH01018C), Ribosomal Protein Lateral Stalk Subunit P0 (*RPLP0*, PPH21138F), RTC (PPX63340A), PPC (PPX63339A), Bone Morphogenetic Protein 6 (*BMP6*, PPH00542F), Interleukin 1 Beta (*IL1B*, PPH00171C) and Vascular Endothelial Growth Factor A (*VEGFA*, PPH00251C).

### 2.5. Data Analysis

The efficiency of the PCR amplification process was evaluated by scoring the stability of PPC Ct values, which were extremely reproducible (18.65 ± 0.36, mean ± SD) across all samples. Due to the stable values, PPC Ct values were used for the initial technical normalization of the other Ct amplification scores. Then, the efficiency of the reverse transcription process was evaluated by scoring the stability of RTC Ct values, which were, again, fairly consistent (22.62 ± 0.31, mean ± SD) across all samples. Those values were used as a second technical normalization step to obtain final Ct values for stability analysis.

HKGs’ stability was tested on the final Ct values with four algorithms: comparative ΔCt method [17], BestKeeper [18], NormFinder [19] and geNorm [20]. Each algorithm used different variables to identify HKGs candidates’ stability. In the ΔCt approach, “pairs of genes” are compared. BestKeeper uses standard deviation (SD), with a higher SD indicating a less stably expressed candidate. Normfinder relies on linear-scale quantitative data, allowing for the identification of a stability value that, when low, indicates high stability. Eventually, geNorm provides an M-value based on the average pairwise expression ratio, with M < 1.5 considered as burden for stability. Each approach generated an HKG stability ranking, with a series of continuous integers starting from 1. The four rankings were compared using RefFinder, a web-based comprehensive tool that assigns an appropriate weight to an individual HKG by calculating the geometric mean for the overall final ranking [21].

### 2.6. Principal Component Analysis and Hierarchical Clustering

Principal component analysis (PCA) and hierarchical clustering of the final Ct values were performed with the ClustVis Beta version webtool (https://biit.cs.ut.ee/clustvis/) [22] under the following parameters: (i) data pre-processing with no transformation, centring and scaling; (ii) heat map with correlation as clustering distance for rows and columns, with average as clustering method for rows and columns and with tighter cluster first for tree ordering for rows and columns.

### 2.7. Statistical Analyses

GraphPad Prism Software v8.0.2 (GraphPad, San Diego, CA, USA) was used for statistical analysis. Shapiro–Wilk normality test (α of 0.01) was performed to test for a normal-data distribution. When the normality test was passed, for analysis between couple of conditions an unpaired parametric Student’s *t*-test was performed; when all conditions were analysed together, an unpaired one-way ANOVA with Tukey post hoc test was used. The level of significance was set at *p*-value ≤ 0.05, with tendency at 0.1 ≤ *p*-value < 0.05.

## 3. Results

### 3.1. ASCs Characterization

**Figure 1:**
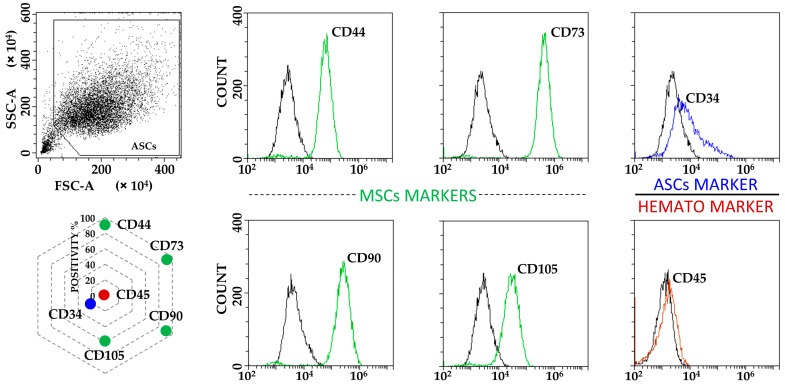
ASCs immunophenotype characterization. ASCs were positive for MSCs markers CD44, CD73, CD90 and CD105, and to tissue resident/early passage ASCs marker CD34. CD45 haematological marker was negative. Plots from a representative donor are shown with black lines for unstained samples and coloured lines for respective Ab-stained ones. Radar chart shows the mean of the three independent donors used in the study for each analysed marker. SD was always ≤10%.

ASCs exhibited typical MSC fibroblast-like morphology (Figure S1), with flow cytometry confirming the standard MSC phenotype given by the positive expression of CD44, CD73, CD90 and CD105 markers, in addition to the absence of haematological epitope CD45. Of note, CD34 was detectable (Figure 1), as previously reported for ASCs in both early passages and in native adipose tissue being gradually lost upon expansion in vitro [23]. Neither starvation nor inflammation had detrimental effects on cell viability, in all cases being ≥95% (median, N = 3): 99 (F), 98 (F+IL1), 98 (S), 99 (S+IL1) and 95 (F+IL1/S).

### 3.2. Expression of Candidate HKGs

**Figure 2:**
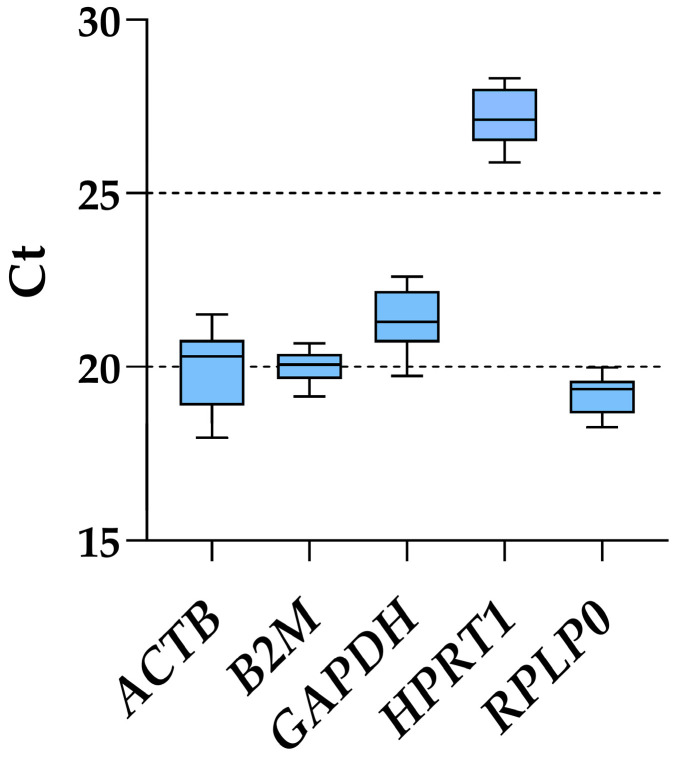
Ct values of tested housekeeping genes across all ASCs samples. Box and whiskers plot is shown, with min to max for whiskers.

Considering all ASC samples, regardless of inflammatory priming or starvation, *RPLP0* had the lowest Ct values and, therefore, the highest amount (mean Ct across all conditions of 19.20 ± 0.53), whereas *HPRT1* was the candidate with the most reduced presence (27.11 ± 0.71) (Figure 2 and Table 1). *ACTB* was the gene with the highest fluctuation between the lowest and highest Ct values, with a delta of 3.54, while *B2M* and *RPLP0* had the smallest (1.53 and 1.73, respectively). This was mirrored by the ranking of the standard deviation values: *B2M* (0.44), *RPLP0* (0.53), *HPRT1* (0.71), *GAPDH* (0.81) and *ACTB* (1.03). Of note, the results of the five genes under study were not influenced by co-regulation in their expression, since *ACTB* is located on chromosome (chr) 7, *B2M* on chr15, *HPRT1* on chrX, and *GAPDH*/*RPLP0* at the opposite extremities on chr12, unlikely belonging to the same cluster [24].

**Figure 3:**
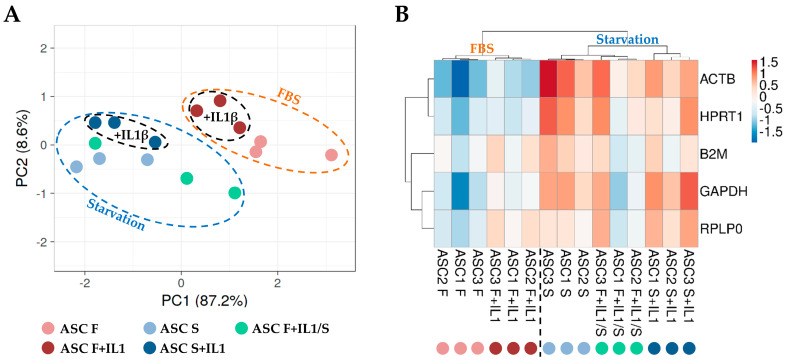
Principal component analysis (PCA) and heat map of HGK expression values. (**A**) In PCA, the X- and Y-axes show principal component 1 and principal component 2, which explain 87.2% and 8.6% of the total variance, respectively. (**B**) In the heat map, positive values mean higher Ct (lower amount), and negative values mean lower Ct (higher amount) with respect to mean values after row centring for each HGK. Both rows and columns were clustered using correlation distance and average linkage. No transformation and no scaling were applied for the dataset.

To score the overall differences in the HKG expression between the different culture conditions, principal component analysis (PCA) and hierarchical clustering approaches were used to analyse the samples (Figure 3). Both analyses clearly emphasized that samples in starvation, regardless of the presence of inflammation, clustered separately from those in the presence of FBS (F and F+IL1 vs. S and S+IL1 and F+IL1/S). Under this dichotomy, the presence of interleukin 1β was able to further divide the conditions (F vs. F+IL1, F+IL1/S and S vs. S+IL1). This was emphasized by the higher similarity for the six samples in starvation without IL1β (F+IL1/S and S) regardless of its previous presence during FBS growth, with respect to those in starvation with the concomitant presence of the inflammatory stimulus (S+IL1). Thus, starvation appeared to overcome inflammation as a discriminant for HKG stability analysis.

### 3.3. HKGs Stability Analysis

In the wake of the preliminary analysis of HKG expression, a stability assessment was performed with four different computational approaches. Since differences in single-applets rankings emerged, integration and normalization of the data were applied, generating a comprehensive geomean value. The first analysis was conducted on the different conditions separately (Table 2). For samples in FBS (F), *HPRT1* resulted the most stable (geomean of 1.19), while *GAPDH* the least (4.23). The situation changed in the presence of inflammation (F+IL1), where *GAPDH* was the best (1.41) and *ACTB* an unreliable candidate (4.73). For ASCs in starvation (S), *B2M* was at the top of the ranking (1.73), and *ACTB*, again, performed poorly (5.00). When inflammation was added during starvation (S+IL1), *RPLP0* emerged as the best HKG (1.19), and *HPRT1* underperformed (5.00). Eventually, for samples in starvation after inflammation (F+IL1/S), *HPRT1* was the most stable (1.73) while *GAPDH* did not show a reliable behaviour (5.00). Thus, it clearly emerges that HGK stability and choice are fairly dependent on the specific culturing condition.

Since in the PCA and clustering analyses clearly showed the primary influence of starvation and the secondary effect of inflammation on HGK modulation, combinatorial tests were performed (Table 3). First, the samples in FBS without (F) or with (F+IL1) IL1β were analysed together. *B2M* (geomean of 1.57) was the best performer and *RPLP0* (4.23) the worst. Of note, identical ranking emerged for samples in starvation without (S) or with (S+IL1) IL1β, with *B2M* (1.32) and *RPLP0* (3.98) being the two opposites. Second, gathering the S and F+IL1/S conditions, *HPRT1* (1.41) ranked first while *GAPDH* (4.23) last. Third, the stability of HKGs in the presence of IL1β (F+IL1 and S+IL1) was tested. *GAPDH* (1.41) was the most reliable candidate, with *B2M* (3.98) acting poorly. Moreover, since often for clinical application envisioned at the use of secretomes ASCs are cultivated in the presence of serum, without or with inflammatory priming, and then starved to collect the released factors and EVs; other two coupled conditions were analysed. When F and S were tested together, *HPRT1* (1.73) emerged as the most stable and *ACTB* (5.00) the least. *ACTB* (5.00) was, again, a poor performer when ASCs were primed before starvation (F+IL1 and F+IL1/S), while *GAPDH* (1.73) was the most reliable. Therefore, as found in the analyses of the single and specific conditions taken separately, the general presence of serum, starvation or inflammation, or even the change from a condition to another one, may greatly affect the reliability of the different HKGs.

Eventually, all conditions were analysed together (Table 4). Overall, *HPRT1* ranked best (geomean of 1.86) and *ACTB* (5.00) last.

### 3.4. Impact of HKG Choice

**Figure 4:**
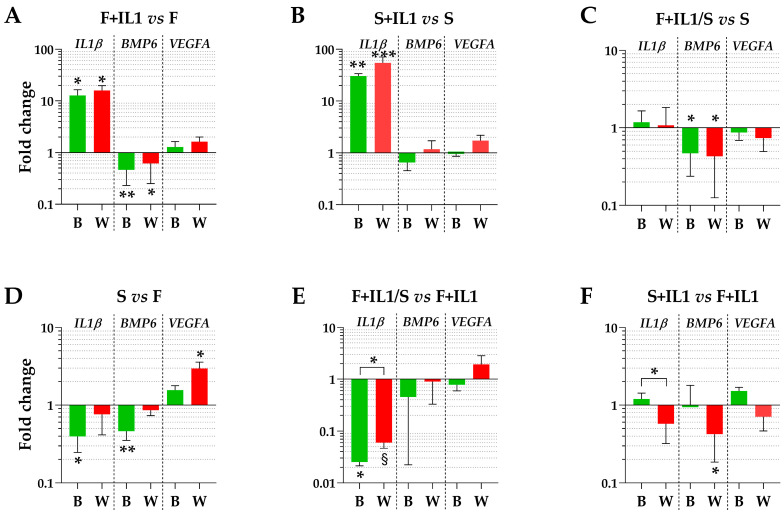
Gene expression modulation is dependent on HKG choice. (**A**–**F**) for *IL1β*, *BMP6* and *VEGFA*, different amounts between treatments may significantly change using the best (B) or worst (W) HKG for each analysed couple. Values are expressed as fold-change mean ± SD, n = 3 independent cell isolates; § for *p*-value ≤ 0.1, * for *p*-value ≤ 0.05, ** for *p*-value ≤ 0.01, *** for *p*-value ≤ 0.001.

The expression levels of three genes (*IL1β*, *BMP6* and *VEGFA*), whose soluble products are involved in OA phenotype at different levels, were tested using the best (B) or the worst (W) HKGs identified in Table 3. Regarding the conditions defined by ASCs cultured in FBS (F) and the counterpart under inflammatory stimuli (F+IL1), the presence of IL1β was able to significantly upregulate its own expression and reduce the *BMP6* amount (fold change ≥ 2 or ≤ 0.5 and *p*-value ≤ 0.05, Figure 4A), while *VEGFA* did not show modulation. A comparable *IL1β* modulation also emerged for the identical comparison in starvation (S+IL1 vs. S) (Figure 4B), while *BMP6* and *VEGFA* did not change. Of note, the best and worst HKGs had similar results, with the loss of a point of significance for *BMP6* for FBS cultured cells and the gain of a point of significance for *IL1β* for starved ASCs. Similarly, for ASCs cultured in starvation regardless of the absence (S) or presence of IL1β (F+IL1/S) during expansion with FBS, the analysis conducted with the two opposite HKGs did not result in significant differences (Figure 4C), with *BMP6* reduced in its expression for cells pre-cultured with IL1β. The situation changed for the comparison of cells cultured in FBS (F) and starvation (S) (Figure 4D). The use of the most reliable HKG allowed to detect a significant downregulation of both *IL1β* and *BMP6* under starvation, that disappeared with the worst HKG. Conversely, *VEGFA* passed from unchanged to apparently significantly altered. In a similar comparison, when inflamed samples in FBS (F+IL1) were considered as milestone, *IL1β* was downregulated in starvation (F+IL1/S) with the best HKG, while with the worst one, it lost its significance (Figure 4E). Again, the use of the less stable HKG suggested an apparent and albeit not significant upregulation of *VEGFA*. Eventually, the presence of IL1β in either FBS (F+IL1) or starvation (S+IL1) never resulted in gene expression differences with the best HKG, while with the worst reference gene, *BMP6* appeared significantly downregulated in the absence of serum (Figure 4F).

**Figure 5:**
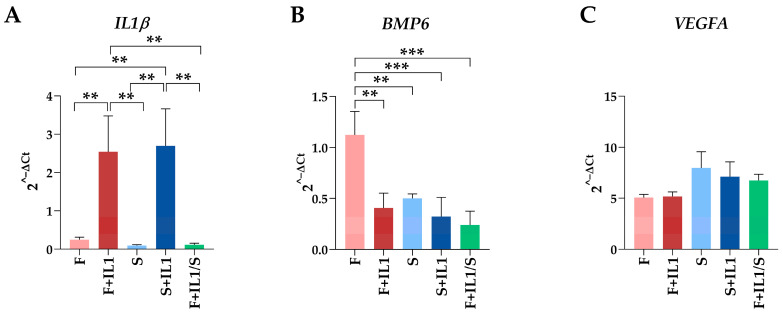
Comparison of gene expression between the five conditions analysed. Gene expression values for *IL1β* (**A**), *BMP6* (**B**) and *VEGFA* (**C**) during the different treatments expressed as 2^−ΔCt^ with respect to the best HKG in Table 4, *HPRT1*. Values are expressed as fold-change mean ± SD, n = 3 independent cell isolates; ** for *p*-value ≤ 0.01, *** for *p*-value ≤ 0.001. For ease of readability, only statistically significant (*p*-value ≤ 0.05) comparisons are indicated. Where no bar is shown, the absence of significance is meant. For *VEGFA*, no comparisons reached statistical significance, making them, therefore, all “not significant”.

Lastly, for each gene, the five conditions were compared together using the best HKGs in Table 4, *HPRT1*. For *IL1β*, inflammation led to its upregulation regardless of the presence or absence of serum (Figure 5A). Of note, starvation after inflammation was able to completely abolish mRNA increase. Also, the significant downregulation observed for S vs. F when analysed with the specific HKG for this couple (Table 3) was lost. Regarding *BMP6*, both inflammation and starvation led to a significant decrease with respect to cells cultured in FBS (F) (Figure 5B). Again, one comparison (F+IL1/S vs. S) lost significance with respect to the analysis performed using the best HKG for the specific couple. Eventually, for *VEGFA*, no differences between conditions emerged (Figure 5C), confirming the data obtained when the single couples were separately analysed with the best specific HKG.

## 4. Discussion

In this work, a rigorous computational approach was used to identify the most stable HKGs in adipose MSCs under inflammatory priming or after starvation. The results highlighted the high variability between conditions, suggesting that each treatment should be considered as stand-alone, and that any comparison should take into account this variability.

MSCs, as bioactive components of orthobiologics or as expanded products, have gained popularity for the treatment of several musculoskeletal conditions, including OA [25]. A major concern is how these cells interact with the joint environment, often inflamed [26], and how this environment can change their features. It has, in fact, been recognized that MSCs may modulate their regenerative and anti-inflammatory activities when in contact with pro-inflammatory conditions [27], both natural and induced. In this regard, to foster MSCs’ therapeutic effects, strategies mimicking what happens when cells are in contact with OA-patients’ synovial fluid have been developed [28], also relying on cell priming through OA-like pro-inflammatory stimuli [29]. Under this approach, it has been possible to obtain either MSCs [30] or MSC-secretome [28,29] with increased anti-inflammatory and chondro-protective potential. As an example, a recent report suggested that IL-1β-primed ASCs increased the phagocytic capacity of neutrophils, which may contribute to inflammation resolution, removal of tissue debris, and support of tissue repair [31], which are all processes required for OA treatment. The main pitfall for translating these approaches to the clinical practice is a direct comparison of outcomes to decipher the most useful protocol. From this perspective, reliable HKGs are mandatory. A bunch of studies in the literature have already proposed the most stable reference genes for MSCs and ASCs under standard culturing conditions [13,14,15], with particular focus on proliferation [14,32], differentiation [14,32,33] and hypoxia [14]. However, to the best of our knowledge, a proposal of HKGs for cells cultivated under OA-like inflammatory conditions is missing, being aware of the array of inflammatory stimuli proposed in the literature [34]. In this report, using IL1β as a stimulating cytokine, *GAPDH* and *RPLP0* always emerged as the two best HKGs in ASCs cultivated in either the presence (F+IL1) or absence (S+IL1) of serum. Of note, without inflammation, both HKGs underperformed in the presence of FBS (F), where *HPRT1* was the most stable, while it had an acceptable stability in starvation (S), resulting in second and third positions in the ranking after *B2M*. Eventually, when directly comparing cells with IL1β without and with FBS (F+IL1 and S+IL1), *GAPDH* emerged as the most stable. The good performance of *GAPDH* under inflammation was further confirmed by its second position in the ranking after *B2M*, considering F and F+IL1 together.

These results suggested that, alongside inflammation, starvation, an essential step in the secretome-production strategy where serum (either bovine or human) contamination has to be avoided, had a great effect on HKGs’ stability. Of note, Figure 3 clearly shows that its influence was even superior to IL1β, with samples first separated based on the presence of serum and then based on the inflammatory stimulus. Under this paradigm, the heatmap further suggests that each donor may have subtle differences in the sensitivity to the different stimuli, as highlighted by the condition-dependent donor alignment, although a deeper analysis with a higher number of genes is needed to shed light on the molecular basis of this donor-related behaviour within the inflammation or starvation dichotomy of samples. To date, starvation is the preferred strategy for secretome/EVs while waiting for next-generation and clinical-grade, chemically defined serum- and xeno-free media [35] that show various influences and should be carefully examined prior to being routinely used for MSC cultures [36]. Starvation alone is usually performed after expansion in FBS or FBS with priming stimuli when the whole secretome or EVs are collected at the price of possible inflammation reduction caused by cytokine removal, while starvation supplemented with inflammation may be envisioned when only EVs at their maximum empowerment have to be produced. A major issue that is often underestimated is the effect of starvation on the cells [37,38,39], often leading researchers to attribute the same features to FBS-fed and starved cells. For this reason, a direct comparison of cells cultured in FBS or starvation is mandatory, and the selection of the most appropriate HKG is crucial. A direct comparison of cells cultured in FBS or starved suggested *HPRT1* as the most stable. Importantly, cultivating cells in the presence of IL1β before starvation (F+IL1 and F+IL1/S) completely changed the ranking, with *GAPDH* as the best performer. Lastly, in cells cultivated in starvation independently of their pre-expansion in the absence or presence of serum (S and F+IL1/S), *HPRT1* emerged as the one with the best performance. The crucial role of *HPRT1* was further emphasized by its high stability when all samples were analysed together. In fact, this “starvation” HKG outperformed the “inflammation” HKG *GAPDH*.

Finally, the misleading effects of a biased selection of the HKGs were evident in the gene expression analysis of three genes related to the OA phenotype and whose amount released by ASCs might influence their, or their secretome, therapeutic potential. As previously discussed, IL1β is a major pro-inflammatory cytokine involved in joint inflammation [40]. BMP6 is actively involved in chondrocyte metabolism, since it promotes the chondrocyte differentiation of progenitor cells [41]. VEGFA is associated with catabolic processes in chondrocytes and synovial cells [42]. Gene expression analyses with the best and worst HKGs for each couple of conditions confirmed the prevailing effects of starvation on the selection of the best performing normalizer with respect to inflammation. In fact, comparing starved and FBS-fed cells clearly showed that the less stable HKGs allowed for apparent and misleading loss (*IL1β* and *BMP6* for S vs. F, *IL1β* for F+IL1/S vs. F+IL1) or gain (*VEGFA* for S vs. F, *BMP6* for S+IL1 vs. F+IL1) of significance. Concerning couples having in common the absence/presence of inflammation, only a point of significance was lost for *BMP6* for F+IL1 vs. F and gained for *IL1β* for S+IL1 vs. S.

The present study had some limitations. The number of HKGs was limited, since many others are reported in the literature and might be tested. We opted for those presented herein for their documented use and availability across several laboratories. Moreover, different media and inflammatory stimuli might be applied to MSCs, therefore increasing the array of reliable and conditions-specific HKG. Finally, several MSC types are used for both research and clinics, making the situation even more complicated and potentially dependent on specific cell types. Nevertheless, we believe that this report is a first step in shedding light on an often undervalued issue regarding inflammation and starvation for molecular studies on adipose-derived MSCs for both research and clinical use.

## 5. Conclusions

Overall, this report confirms the high variability of HKG stability in adipose-derived MSCs. Both inflammation and starvation strongly affect HKG reliability, with starvation being a potent modulator of reference gene expression. With this work, the authors want to stimulate the scientific community and MSC researchers to identify specific HKGs for the conditions to be tested for both research and clinical applications.

## Figures and Tables

**Table 1:** Ct values of tested housekeeping genes across all ASCs samples.

	*ACTB*	*B2M*	*GAPDH*	*HPRT1*	*RPLP0*
**ASC1 F**	17.96	19.15	19.73	25.90	18.25
**ASC2 F**	18.71	19.78	20.71	26.44	18.54
**ASC3 F**	18.77	19.71	20.55	26.51	18.66
**ASC1 F+IL1**	19.03	19.66	21.02	26.34	19.06
**ASC2 F+IL1**	18.88	20.24	21.17	26.68	19.50
**ASC3 F+IL1**	19.54	20.38	21.29	26.59	19.59
**ASC1 S**	21.09	20.42	22.20	28.01	19.36
**ASC2 S**	20.50	20.07	21.77	27.44	19.06
**ASC3 S**	21.50	20.68	22.18	28.30	19.38
**ASC1 S+IL1**	20.78	20.43	22.33	27.39	19.86
**ASC2 S+IL1**	20.34	19.64	21.96	27.12	19.48
**ASC3 S+IL1**	20.69	20.18	22.59	28.02	19.98
**ASC1 F+IL1/S**	19.90	19.28	20.38	26.71	18.43
**ASC2 F+IL1/S**	20.30	19.58	21.04	27.20	18.90
**ASC3 F+IL1/S**	21.05	20.33	22.13	28.07	19.88

F stands for cells cultivated for 48 h in FBS, F+IL1 for FBS with interleukin 1β, S for cells cultivated for 48 h in starvation, S+IL1 for starvation with interleukin 1β, F+IL1/S for cells cultivated for 48 h in FBS with interleukin 1β and further cultivated for 48 h in starvation.

**Table 2:** Stability rankings of tested HKGs, with each condition analysed separately.

Cond.	Geomean		DeltaCt		BestKeeper		NormFinder		geNorm	
**F**	*HPRT1*	1.19	*HPRT1*	0.14	RPLP0	0.16	*HPRT1*	0.04	*B2M|HPRT1*	0.07
	*B2M*	1.86	*B2M*	0.14	B2M	0.26	*B2M*	0.04		
	*ACTB*	3.22	*ACTB*	0.16	HPRT1	0.26	*ACTB*	0.08	*ACTB*	0.11
	*RPLP0*	3.34	*GAPDH*	0.22	ACTB	0.35	*GAPDH*	0.21	*GAPDH*	0.14
	*GAPDH*	4.23	*RPLP0*	0.22	GAPDH	0.40	*RPLP0*	0.22	*RPLP0*	0.18
**F+IL1**	*GAPDH*	1.41	*RPLP0*	0.18	GAPDH	0.09	*RPLP0*	0.03	*GAPDH|HPRT1*	0.12
	*RPLP0*	1.73	*GAPDH*	0.20	HPRT1	0.13	*GAPDH*	0.06		
	*HPRT1*	2.06	*HPRT1*	0.22	RPLP0	0.22	*HPRT1*	0.15	*RPLP0*	0.14
	*B2M*	4.23	*B2M*	0.24	ACTB	0.26	*B2M*	0.19	*B2M*	0.17
	*ACTB*	4.73	*ACTB*	0.34	B2M	0.29	*ACTB*	0.32	*ACTB*	0.24
**S**	*B2M*	1.73	*B2M*	0.16	RPLP0	0.14	*B2M*	0.07	*GAPDH|RPLP0*	0.07
	*RPLP0*	2.00	*HPRT1*	0.18	GAPDH	0.19	*HPRT1*	0.11		
	*GAPDH*	2.06	*GAPDH*	0.19	B2M	0.21	*GAPDH*	0.14	*B2M*	0.12
	*HPRT1*	2.83	*RPLP0*	0.21	HPRT1	0.32	*RPLP0*	0.19	*HPRT1*	0.17
	*ACTB*	5.00	*ACTB*	0.23	ACTB	0.35	*ACTB*	0.22	*ACTB*	0.19
**S+IL1**	*RPLP0*	1.19	*RPLP0*	0.17	ACTB	0.18	*RPLP0*	0.04	*GAPDH|RPLP0*	0.08
	*GAPDH*	1.86	*GAPDH*	0.18	RPLP0	0.20	*GAPDH*	0.04		
	*ACTB*	2.28	*ACTB*	0.21	GAPDH	0.22	*ACTB*	0.14	*ACTB*	0.12
	*B2M*	4.00	*B2M*	0.28	B2M	0.30	*B2M*	0.25	*B2M*	0.17
	*HPRT1*	5.00	*HPRT1*	0.32	HPRT1	0.34	*HPRT1*	0.31	*HPRT1*	0.23
**F+IL1/S**	*HPRT1*	1.73	*HPRT1*	0.13	B2M	0.40	*HPRT1*	0.03	*ACTB|B2M*	0.06
	*B2M*	2.00	*RPLP0*	0.14	ACTB	0.42	*RPLP0*	0.03		
	*ACTB*	2.06	*ACTB*	0.16	HPRT1	0.50	*ACTB*	0.11	*HPRT1*	0.11
	*RPLP0*	2.83	*B2M*	0.19	RPLP0	0.54	*B2M*	0.18	*RPLP0*	0.12
	*GAPDH*	5.00	*GAPDH*	0.25	GAPDH	0.63	*GAPDH*	0.24	*GAPDH*	0.17

Condition (Cond.): F stands for cells cultivated for 48 h in FBS, F+IL1 for FBS with interleukin 1β, S for cells cultivated for 48 h in starvation, S+IL1 for starvation with interleukin 1β, F+IL1/S for cells cultivated for 48 h in FBS with interleukin 1β and further cultivated for 48 h in starvation. geNorm calculates the couple of best HKGs.

**Table 3:** Stability rankings of tested HKGs; coupled conditions.

Cond.	Geomean		DeltaCt		BestKeeper		NormFinder		geNorm	
**F and F+IL1**	*B2M*	1.57	*B2M*	0.24	*HPRT1*	0.19	*B2M*	0.10	*ACTB|GAPDH*	0.21
	*GAPDH*	2.00	*GAPDH*	0.26	*B2M*	0.33	*GAPDH*	0.17		
	*ACTB*	2.28	*ACTB*	0.28	*ACTB*	0.34	*ACTB*	0.19	*B2M*	0.24
	*HPRT1*	3.34	*RPLP0*	0.29	*GAPDH*	0.42	*RPLP0*	0.21	*RPLP0*	0.25
	*RPLP0*	4.23	*HPRT1*	0.32	*RPLP0*	0.45	*HPRT1*	0.27	*HPRT1*	0.28
**S and S+IL1**	*B2M*	1.32	*B2M*	0.33	*GAPDH*	0.20	*B2M*	0.18	*ACTB|B2M*	0.18
	*GAPDH*	2.00	*GAPDH*	0.34	*B2M*	0.27	*GAPDH*	0.20		
	*ACTB*	2.45	*ACTB*	0.35	*RPLP0*	0.27	*ACTB*	0.26	*HPRT1*	0.24
	*HPRT1*	3.94	*HPRT1*	0.37	*ACTB*	0.32	*HPRT1*	0.28	*GAPDH*	0.32
	*RPLP0*	3.98	*RPLP0*	0.44	*HPRT1*	0.40	*RPLP0*	0.41	*RPLP0*	0.37
**S and F+IL1/S**	*HPRT1*	1.41	*HPRT1*	0.19	*RPLP0*	0.37	*HPRT1*	0.04	*ACTB|HPRT1*	0.08
	*B2M*	2.21	*B2M*	0.21	*B2M*	0.42	*B2M*	0.09		
	*ACTB*	2.28	*ACTB*	0.21	*ACTB*	0.49	*ACTB*	0.13	*B2M*	0.12
	*RPLP0*	3.34	*GAPDH*	0.30	*HPRT1*	0.51	*GAPDH*	0.25	*GAPDH*	0.19
	*GAPDH*	4.23	*RPLP0*	0.32	*GAPDH*	0.60	*RPLP0*	0.28	*RPLP0*	0.25
**F+IL1 and S+IL1**	*GAPDH*	1.41	*GAPDH*	0.38	*RPLP0*	0.23	*GAPDH*	0.16	*GAPDH|HPRT1*	0.17
	*HPRT1*	1.86	*HPRT1*	0.40	*B2M*	0.29	*HPRT1*	0.17		
	*RPLP0*	2.45	*RPLP0*	0.42	*HPRT1*	0.49	*RPLP0*	0.20	*ACTB*	0.29
	*ACTB*	3.94	*ACTB*	0.54	*GAPDH*	0.57	*ACTB*	0.49	*RPLP0*	0.38
	*B2M*	3.98	*B2M*	0.60	*ACTB*	0.73	*B2M*	0.57	*B2M*	0.47
**F and S**	*HPRT1*	1.73	*HPRT1*	0.42	*RPLP0*	0.39	*HPRT1*	0.10	*B2M|RPLP0*	0.15
	*RPLP0*	2.00	*GAPDH*	0.44	*B2M*	0.42	*GAPDH*	0.10		
	*B2M*	2.06	*B2M*	0.51	*HPRT1*	0.82	*B2M*	0.41	HPRT1	0.37
	*GAPDH*	2.83	*RPLP0*	0.55	*GAPDH*	0.86	*RPLP0*	0.49	*GAPDH*	0.39
	*ACTB*	5.00	*ACTB*	0.74	*ACTB*	1.28	*ACTB*	0.72	*ACTB*	0.53
**F+IL1 and F+IL1/S**	*GAPDH*	1.32	*GAPDH*	0.44	*GAPDH*	0.36	*GAPDH*	0.08	*B2M|RPLP0*	0.14
	*RPLP0*	2.06	*RPLP0*	0.47	*B2M*	0.40	*HPRT1*	0.29		
	*B2M*	2.21	*B2M*	0.52	*RPLP0*	0.43	*RPLP0*	0.35	*GAPDH*	0.24
	*HPRT1*	3.36	*HPRT1*	0.52	*HPRT1*	0.47	*B2M*	0.43	*HPRT1*	0.41
	*ACTB*	5.00	*ACTB*	0.73	*ACTB*	0.63	*ACTB*	0.69	*ACTB*	0.53

Condition (Cond.): F stands for cells cultivated for 48 h in FBS, F+IL1 for FBS with interleukin 1β, S for cells cultivated for 48 h in starvation, S+IL1 for starvation with interleukin 1β, F+IL1/S for cells cultivated for 48 h in FBS with interleukin 1β and further cultivated for 48 h in starvation. geNorm calculates the couple of best HKGs.

**Table 4:** Stability rankings of tested HKGs; all conditions analysed together.

Cond.	Geomean		DeltaCt		BestKeeper		NormFinder		geNorm	
**ALL**	*HPRT1*	1.86	*HPRT1*	0.48	*B2M*	0.40	*HPRT1*	0.20	*B2M|RPLP0*	0.34
	*B2M*	2.00	*GAPDH*	0.48	*RPLP0*	0.46	*GAPDH*	0.22		
	*RPLP0*	2.06	*RPLP0*	0.54	*HPRT1*	0.62	*RPLP0*	0.40	*GAPDH*	0.44
	*GAPDH*	2.63	*B2M*	0.57	*GAPDH*	0.71	*B2M*	0.45	*HPRT1*	0.47
	*ACTB*	5.00	*ACTB*	0.66	*ACTB*	0.90	*ACTB*	0.60	*ACTB*	0.54

Condition (Cond.): All stands for all samples analysed together. geNorm calculates the couple of best best HKGs.

## Data Availability

Raw data for this study are available at https://osf.io/4eh8s/?view_only=7fc251d2dfb048d89b3056e931b12a85 (created on 6 November 2023).

## Supplementary Materials

The following supporting information can be downloaded at: https://www.mdpi.com/article/10.3390/cimb46010054/s1, Figure S1: ASC morphology confirming fibroblast-like features.
