# Supplementary figures and images for: Inflammation and Starvation Affect Housekeeping Gene Stability in Adipose Mesenchymal Stromal Cells
Source: Curr Issues Mol Biol. 2024 Jan 19;46(1):842–55. doi: 10.3390/cimb46010054 (PMC10814131; doi:10.3390/cimb46010054)

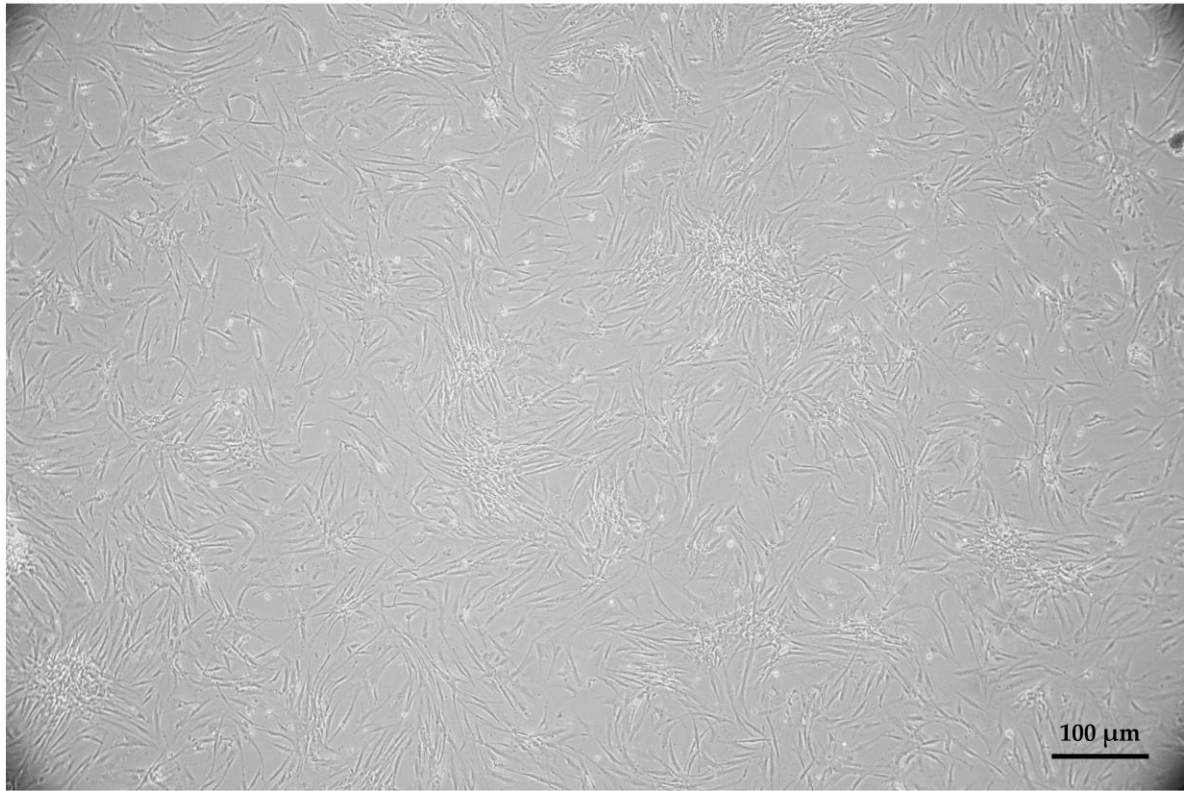

**Figure S1.** ASC morphology confirming fibroblast-like features.

Supplement: Supplementary file 1 [file cimb-46-00054-s001.zip › cimb-2805296-supplementary.pdf]
